# Toward Trustworthy Diabetes Technologies: A Dynamic Platform for Safety and Security Evaluation

**DOI:** 10.34133/bmef.0317

**Published:** 2026-09-30

**Authors:** Anna R. Bird, Vivek Mohan, Kexin Wei, Zong Cao, Shi Hoe Ng, Manasvi M. Mulay, Viktor Schlegel, Miro G. Moffett, James E. Moore, Chip-Hong Chang, Nick Oliver, Anil A. Bharath

**Affiliations:** ^1^ Imperial Global Singapore, Imperial College London, London, UK.; ^2^ School of Electrical and Electronic Engineering, Nanyang Technological University, Singapore, Singapore.; ^3^ Department of Bioengineering, Imperial College London, London, UK.; ^4^ College of Computing and Data Science, Nanyang Technological University, Singapore, Singapore.; ^5^ School of Chemistry, Chemical Engineering, and Biotechnology, Nanyang Technological University, Singapore, Singapore.; ^6^ Department of Computer Science, The University of Manchester, Manchester, UK.; ^7^ Department of Metabolism, Digestion and Reproduction, Imperial College London, London, UK.

## Abstract

**Objective:** To evaluate the cybersecurity of automated insulin delivery (AID) systems, a platform was developed, integrating continuous glucose monitors, fluidic systems, insulin controllers, and wireless communication pathways with virtual subjects. **Impact Statement:** This study provides evidence that AID systems that are vulnerable to cyberattacks pose substantial risk to patient safety. This underscores the need for improved regulation and design of personal, wearable medical devices, through cyberphysiological system modeling. **Introduction:** AID systems depend on continuous glucose monitors (CGMs) as their primary sensing input, making evaluation of the accuracy, robustness, and security of these sensors essential for safe diabetes management. However, there are limited assessment tools that preserve the communication and timing paths of CGM sensors within an AID system. **Methods:** We present a modular hardware-in-the-loop platform that reproduces physiological glucose dynamics, bridging the gap between simulation and clinical validation. The system integrates programmable peristaltic pumps, 3D-printed flow cells, glucose sensors, and a real-time virtual subject simulator. **Results:** The platform generates reproducible glucose trajectories and supports closed-loop operation through integration with an open-source insulin controller. A false data injection attack scenario on the glucose sensor led to an increase in insulin dosage delivered by the control algorithm relative to baseline. If undetected, this could result in a life-threatening condition. **Conclusion:** The platform enables system-level cybersecurity assessment of AID under dynamic operating conditions and highlights safety–security interdependencies in closed-loop medical therapeutics.

## Introduction

Diabetes mellitus is a long-term condition that places a substantial and growing burden on the healthcare system. Forecasts predict a global prevalence of 9.8% by the year 2050 [1], with over 90% of the cases attributable to type 2 diabetes as of 2024 [2]. This escalating burden has driven rapid innovation in technologies that enable people to self-manage diabetes, including continuous glucose monitoring (CGM), insulin pumps, and automated insulin delivery (AID) systems [3]. While the implementation of AID has mostly been limited to the management of type 1 diabetes (T1D), emerging evidence shows that AID can also lead to rapid and sustained improvements in the glycemic control of type 2 diabetes patients [4]. Clinical adoption of AID has been associated with increased time spent in the glucose target range and reduced hypoglycemia—which are associated with improved long-term health outcomes [5]. However, these benefits are critically dependent on reliable sensing, communication, and control across multiple interoperable devices, underscoring the need for improved standardization of device performance [6] and systematic consideration of cybersecurity risks.

The development and validation of CGM and AID algorithms have traditionally followed staged testing pathways. In silico simulation tools such as the UVA/Padova T1D simulator [7] are now well established for testing AID control algorithms, enabling reproducible population-level testing and shortening the path to regulatory clearance. Their utility for control tuning and preclinical safety evaluation is well documented and continues to improve [8]. While computational models are well suited for assessing control algorithms, they cannot capture the physical behavior of wearable sensors, the effects of fluid dynamics and mass transport, or the integrity of wireless communication pathways. Recent work has begun to formalize dynamic in vitro CGM testing protocols that expose sensors to controlled, time-varying analyte concentrations, flow conditions, and interferents [9–11]. Although these studies demonstrate the feasibility of dynamic test beds for robust sensor characterization, they do not assess the interoperability of CGM within a closed-loop AID system.

A standardized physical test bed that reproduces interstitial glucose dynamics in real time, while integrating all elements of an AID system, has several advantages. First, it can substantially accelerate device and algorithm iteration. Second, it may reduce the duration and scale of clinical testing required for each design cycle. Crucially, it allows the safe exploration of edge cases or cybersecurity risk scenarios that would otherwise be unethical, unsafe, or impractical to study in humans. Organ-on-chip platforms [12] that integrate commercial CGM sensors to monitor glucose dynamics have been demonstrated before. Extending these concepts to a hardware-in-the-loop (HIL) framework that combines validated physiological simulators, CGM sensing, and insulin controllers preserves the timing, noise characteristics, and wireless data paths of real devices while enabling deterministic and repeatable experimentation. Such a framework thus enables standardized comparison of devices across manufacturers under controlled yet physiologically realistic conditions.

Wireless communication is a critical dependency in AID systems and a potential point of failure. Compromised or disrupted communication, whether due to technical faults, sensor defects, or cyberattacks, can directly impact insulin dosing and patient safety [13]. Recently, CGM sensors from a specific production line were recalled by their manufacturer due to incorrect low glucose readings, resulting in undetected sustained hyperglycemia over a number of days; 736 severe adverse events and 7 deaths were reported [14]. Wireless links, smartphone relays, cloud services, and controller radios create multiple opportunities for eavesdropping, replay, or message manipulation attacks [15–20]. While passive attacks primarily threaten privacy through unauthorized access to data [21], active communication-layer attacks can alter control signals and destabilize closed-loop operation. Increasing interoperability has expanded the cybersecurity attack surface of AID systems, a concern highlighted in recent regulatory guidance [22]. These risks are not hypothetical: prior security research and public advisories have demonstrated exploitable vulnerabilities in commercially available diabetes devices, including insulin pump systems with insecure radio protocols and insufficient authentication [23,24]. Such findings underscore the need to evaluate not only sensor accuracy and algorithmic performance but also the integrity and resilience of the cyberphysical feedback loop in AID systems [9,20].

Testing the resilience of AID to cybersecurity threats is a challenge in clinical settings, as the consequences of the test scenarios could have severe implications. This further underscores the need for dynamic HIL test systems that simultaneously reproduce physiological glucose dynamics and preserve the system’s communication and control chain. Such a rig enables (a) the generation of realistic glucose profiles with dynamic changes based on real-time feedback from a virtual subject glucose–insulin response simulator and (b) the controlled simulation of system failures or cybersecurity attacks. By bridging in silico algorithm validation, benchtop sensor testing, and wireless device integration, a modular HIL test rig can support early-stage safety assessment, facilitate the development of protective mechanisms, and provide regulators with a reproducible framework for evaluating robustness and cyber-resilience prior to—and in parallel with—clinical testing.

Here, we present a test rig (Fig. 1) that integrates physical glucose sensors with simulated virtual subjects from a validated database [7]. The key innovations of this system are the preservation of real-world dynamics and the closed-loop modeling of sensor hardware with virtual subject simulators and insulin control algorithms. Virtual subject glucose levels, determined by a real-time glucose–insulin response simulator, are reproduced in the real world by pulling glucose and buffer solutions in varied ratios from reservoirs. Peristaltic pumps deliver this glucose solution to a CGM fitted into a fluidic system. The glucose value is updated once per minute to match the maximum sampling frequency of commercial devices. The readings from the CGM are used to drive the insulin controller and virtual subject simulator, resulting in a model closed-loop system. We demonstrate the utility of this hybrid platform for testing the resilience of AID systems to cyberthreats.

**Fig. 1.**
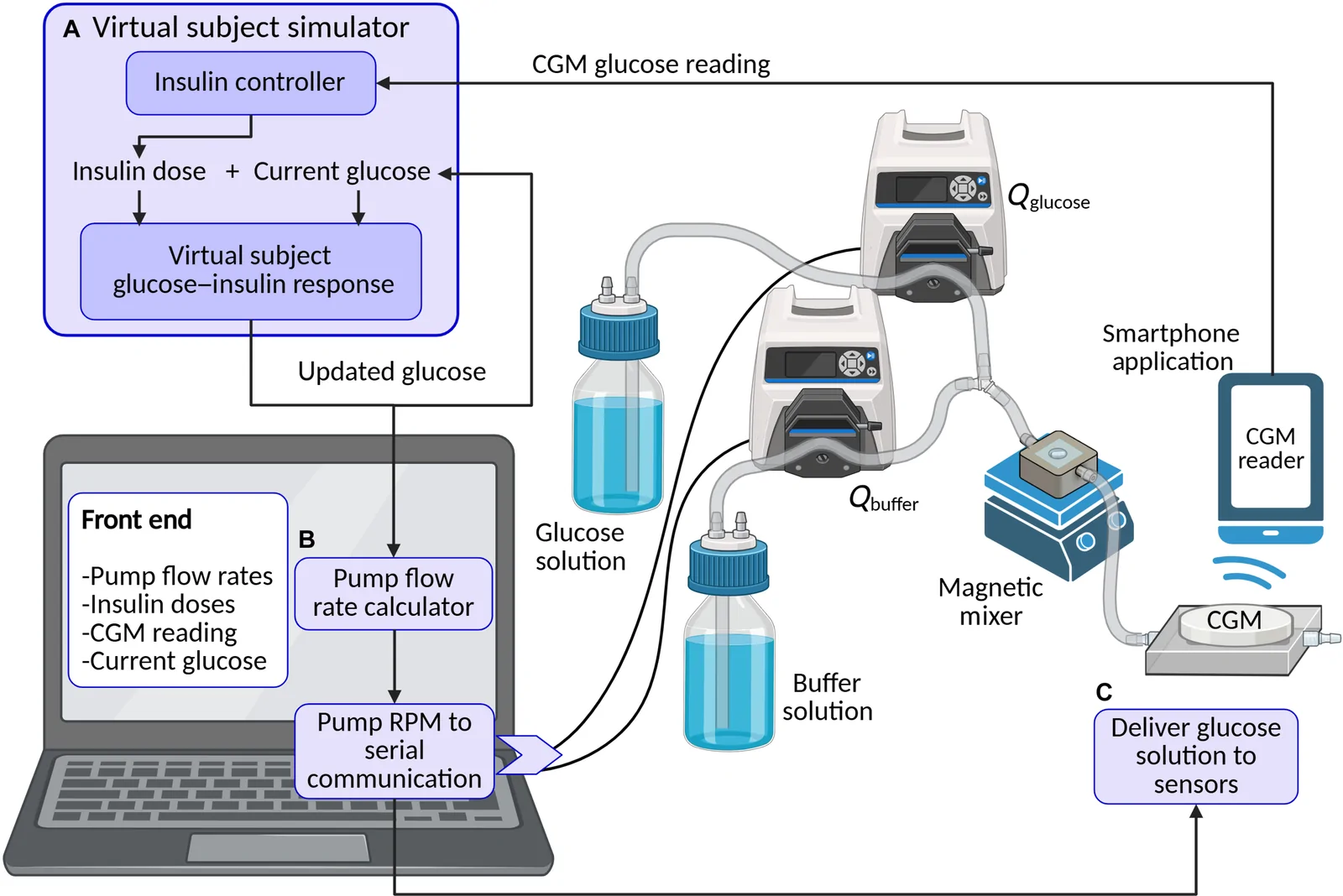
Schematic of the dynamic test rig. (A) A virtual subject simulator updates the glucose value, which is passed to the system front end. (B) Peristaltic pumps connected to glucose and buffer reservoirs deliver changing glucose concentration to a flow cell with an attached continuous glucose monitor (CGM). (C) CGM data are transmitted via a smartphone to the insulin controller. The calculated insulin dose is passed to the real-time virtual subject glucose–insulin response simulator, which iteratively updates glucose predictions and modulates pump rates to replicate interstitial glucose dynamics.

## Results

### Virtual subject glucose response

To generate physiologically realistic glucose dynamics, the UVA/Padova T1D simulator was adapted to produce simulation results at a 1-min CGM sampling frequency, instead of simulating the glucose trajectories for a fixed interval (e.g., 24 h). This matches the sampling frequency of CGM and creates a virtual subject, rather than a data generator for testing in silico treatment. Across 10 child and 20 adult virtual participants, with 3 meal scenarios each, the real-time implementation closely matched the reference UVA/Padova simulator. The root mean squared error (RMSE) was 0.036 ± 0.02 mmol/l for child subjects (*N* = 10 participants, *n* = 3 meal scenarios per participant) and 0.022 ± 0.013 mmol/l for adult subjects (*N* = 20, *n* = 3) (Fig. 2A). These errors are small relative to the physiological ranges of glucose (3 to 20 mmol/l) and the maximum resolution of available CGM sensors (0.1 mmol/l). Agreement was further quantified using the mean absolute relative difference (MARD), a measure of average difference between device measurement and reference [25]. The MARD between the real-time implementation and the reference simulator was 0.19% ± 0.11% for child subjects and 0.14% ± 0.09% for adult subjects (Fig. 2B), well below typical CGM performance thresholds (10% to 20%) [26].

**Fig. 2.**
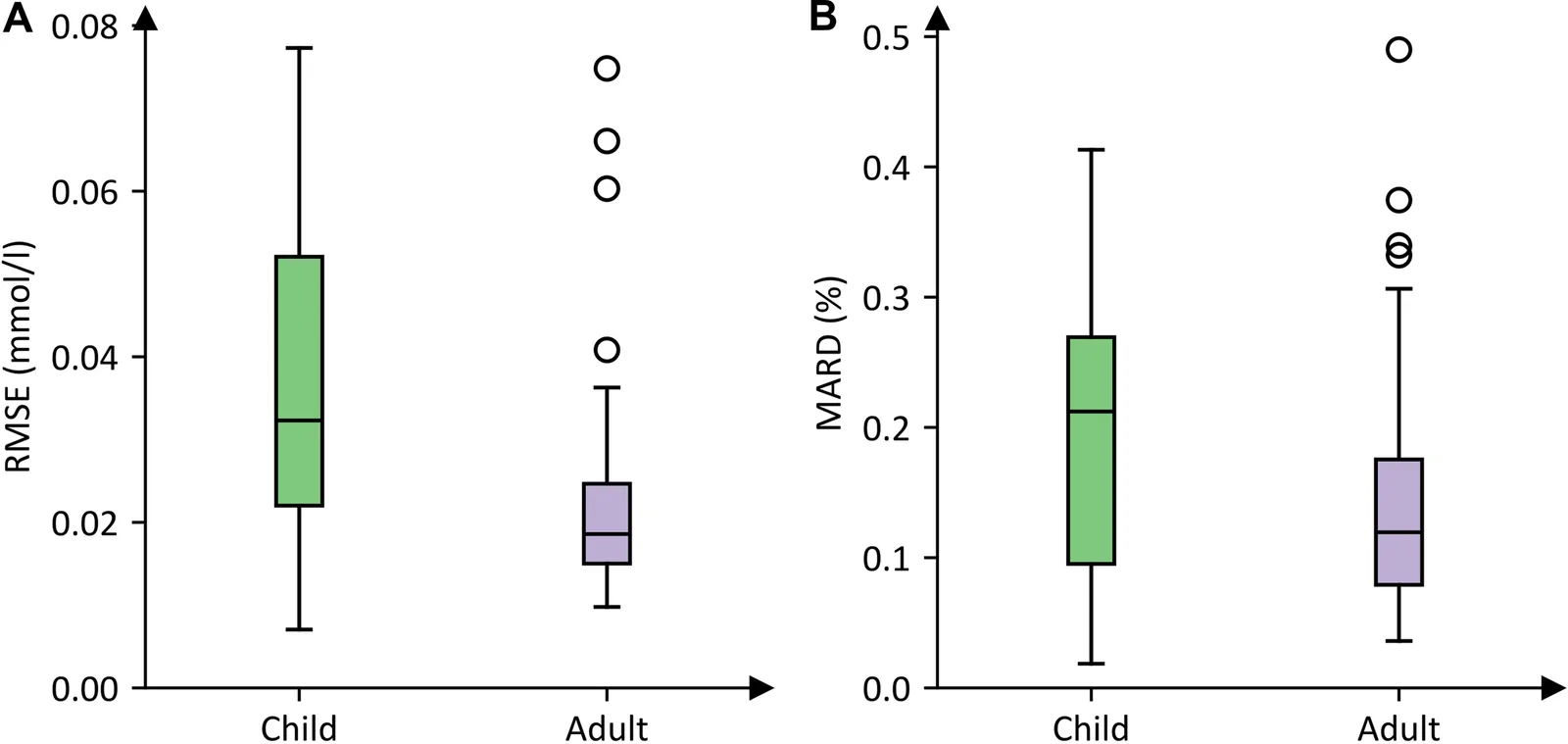
Comparison between glucose data from the real-time glucose–insulin response simulator and the UVA/Padova model for child and adult virtual subjects. (A) Root mean squared error (RMSE) and (B) mean absolute relative difference (MARD).

### Flow system validation

To reproduce the simulated glucose trajectories in the real world, a fluidic system was built, using peristaltic pumps to pull from glucose solution and buffer solution reservoirs in varying ratios. Dynamic glucose modulation within the fluidic test rig was validated using glucose-sensitive screen-printed electrodes (SPEs) fitted into custom flow cells. Under a constant glucose concentration (6 mmol/l), the SPE response exhibited flow-rate dependence, with a reduced current under static conditions and a modest increase in signal magnitude with increasing flow rate (Fig. 3A). Based on these measurements, a total flow rate of 47.5 mm^3^/s was selected for subsequent experiments.

**Fig. 3.**
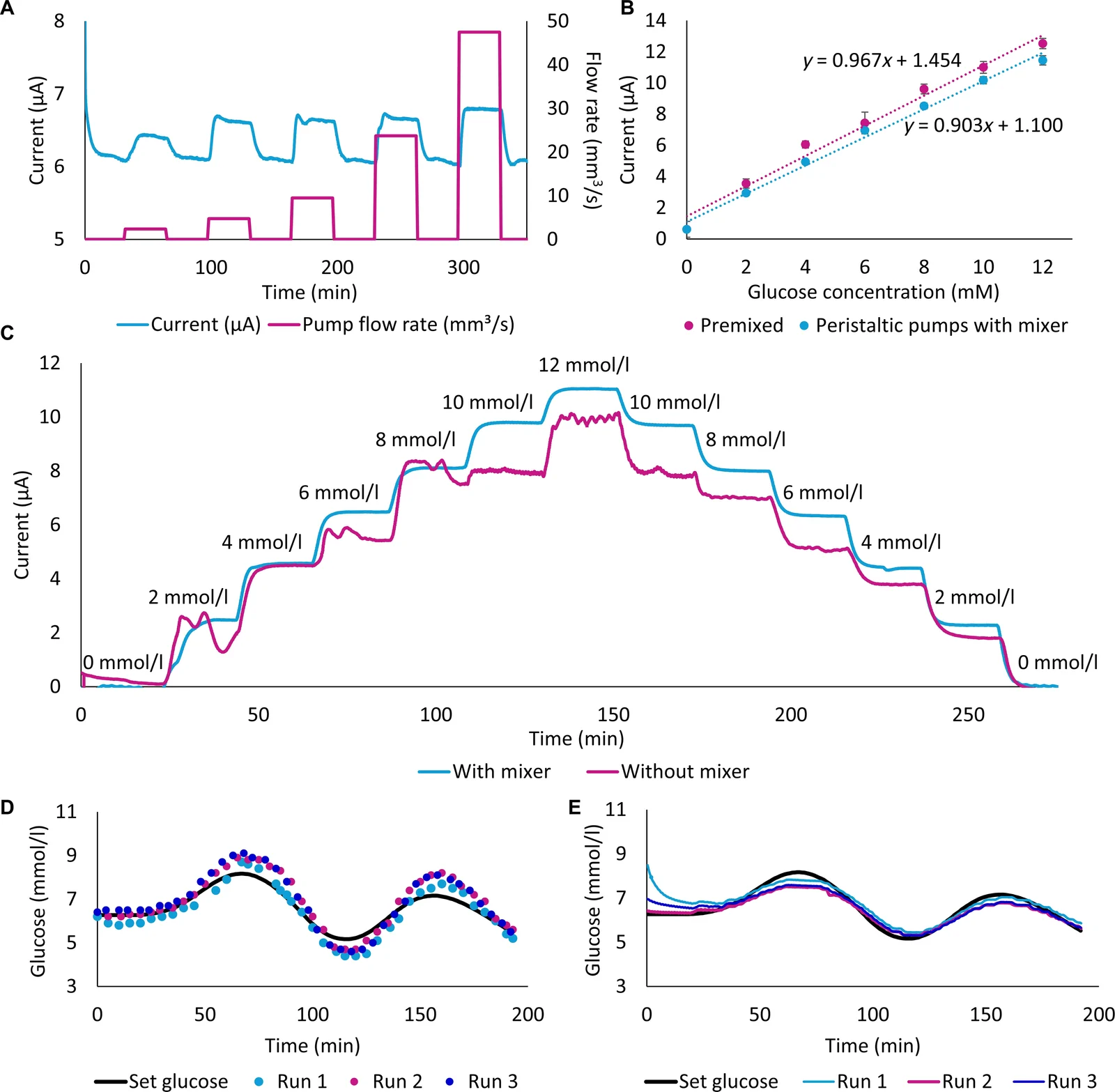
Flow system validation. (A) Response of the screen-printed electrode (SPE) glucose sensor to premixed 6 mmol/l glucose solution passed through the test rig flow cell at different flow rates. (B) Calibration plot of the SPE sensor in premixed glucose solutions (0 to 12 mmol/l) flown through the test rig flow cell at *Q* = 47.5 mm^3^/s and the same concentrations generated by pulling solution from buffer and glucose reservoirs and modulating the flow rate of respective peristaltic pumps while maintaining a constant *Q*_total_ = 47.5 mm^3^/s. (C) SPE glucose sensor response to programmed glucose concentrations, with and without the mixing chamber preceding the flow cell. Glucose readings from (D) a commercial continuous glucose monitor (CGM) and (E) an in-house SPE sensor in response to a preprogrammed glucose profile. The commercial CGM and SPE sensors were connected in series, and 3 replicates were performed. The programmed glucose value is shown as a solid black line. CGM readings are shown as dots, and SPE readings are shown as lines, with colors corresponding to the same replicate.

At this flow rate, SPE sensors demonstrated responsiveness to changes in glucose concentration, with a linear range from 0 to 12 mmol/l. Using premixed glucose solutions delivered by a single peristaltic pump, the measured sensitivity was 0.97 μA/mM (Fig. 3B). Comparable sensitivity (0.903 μA/mM) was obtained when glucose concentrations were generated dynamically by modulating the relative flow rates of glucose and buffer from reservoirs while maintaining constant total flow. Incorporation of a magnetic mixing compartment upstream of the flow cell improved signal stability and reproducibility (Fig. 3C). The results indicate a similar response time regardless of whether the concentration was increased or decreased. The temporal response of the flow system was assessed by stepwise changes in glucose concentration. The response time, defined as the onset of detectable change in sensor signal, was 2.43 ± 0.40 min for increasing glucose concentration (*n* = 4) and 2.41 ± 0.51 min for decreasing glucose concentration (*n* = 3) (Fig. S1).

The system was further evaluated using physiologically relevant glucose profiles generated by the real-time glucose–insulin response simulator. These profiles were preprogrammed into the pump controller and delivered to sensors arranged in series, consisting of a commercial CGM sensor followed by an in-house SPE sensor. Both sensors were responsive and reproduced the programmed glucose trajectory with minute-level resolution across 3 replicates (Fig. 3D and E). Using the programmed glucose concentration as reference, the MARD for the commercial CGM sensor was 5.6%, 6.3%, and 7.0%, while the SPE exhibited MARD values of 5.0%, 3.2%, and 3.9% for runs 1, 2, and 3, respectively.

### Glucose regulation with an insulin controller

To complete the AID loop, an insulin controller was deployed in conjunction with the real-time glucose–insulin response simulator. CGM sensor data, together with predefined virtual participant parameters and the amount of carbohydrates (CHO) ingested at meals, are inputs to the controller, used to inform insulin-dosing decisions. The test rig utilizes an open-source artificial pancreas system algorithm (OpenAPS) (available at https://github.com/openaps/oref0) as the insulin controller. Glycemic outcomes were compared against the standard basal–bolus controller built into the UVA/Padova model under identical meal scenarios.

Following a 75-g carbohydrate meal, insulin boluses delivered by the OpenAPS meal wizard were comparable to those calculated by the basal–bolus controller (approximately 6 U). Overall glycemic control is quantified by the percentage of time that a patient’s blood glucose is within defined target ranges (indicated by the green shaded band in Fig. 4). These parameters are time in range (TIR), time above range (hyperglycemia, TAR), and time below range (hypoglycemia, TBR). Usage of the basal–bolus controller led to 11.2% TAR, 88.8% TIR, and no TBR; using OpenAPS resulted in 12.8% TAR, 87.2% TIR, and no TBR (Fig. 4). Both controllers exceeded the recommended threshold of 70% TIR [27].

**Fig. 4.**
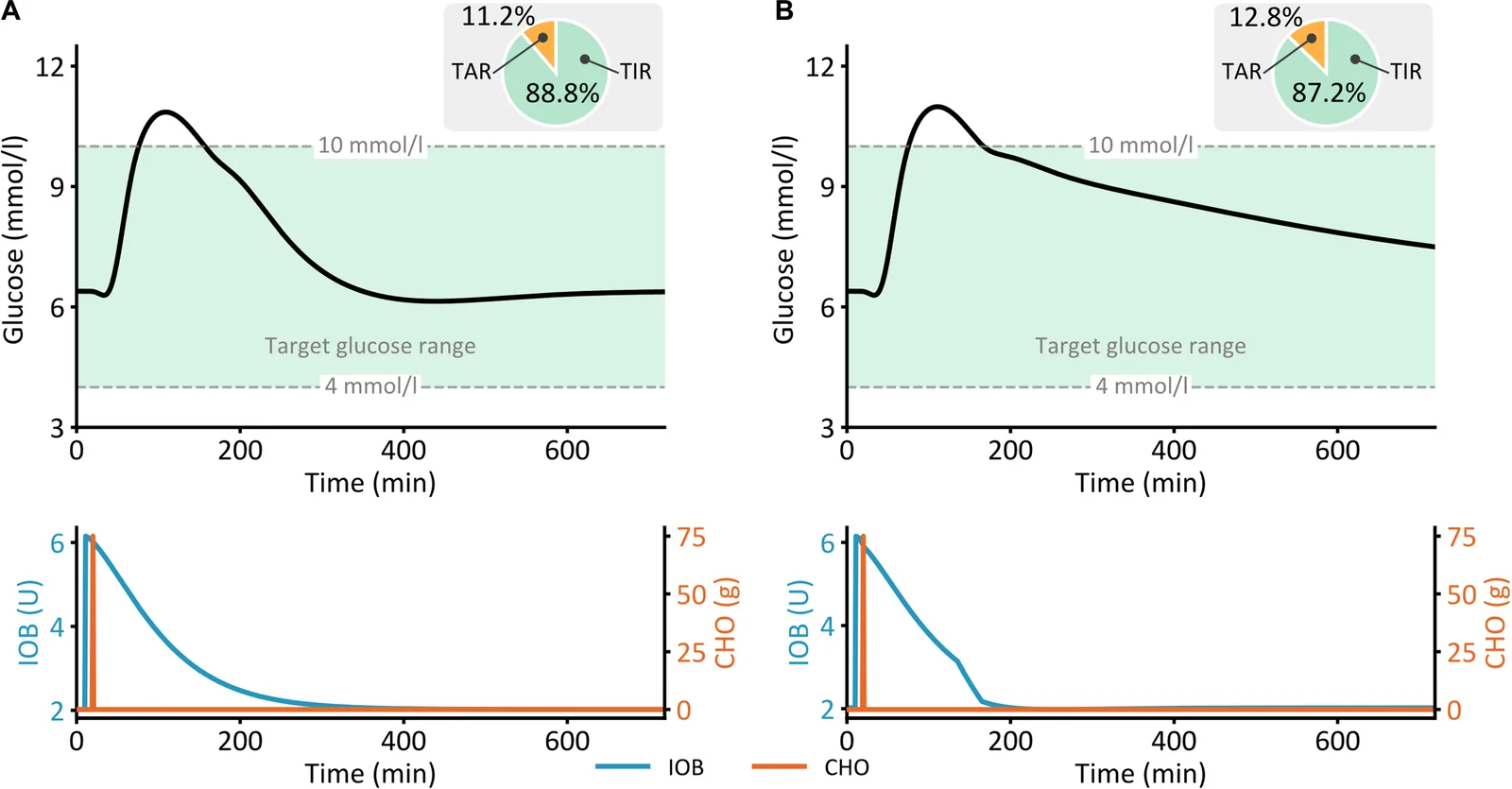
Virtual subject model simulation results. Simulated 75-g meal at 20 min, with insulin on board (IOB) from either (A) in-built basal–bolus controller from the UVA/Padova model, with 11.2% time above range (TAR) and 88.8% time in range (TIR) or (B) implementation of OpenAPS, with 12.8% TAR and 87.2% TIR.

### Simulated hybrid closed loop and cybersecurity attack

With validated flow performance and integrated physiological simulation and insulin control, the test rig was operated in a hybrid closed-loop setting with simulated meals. Dynamically modulated glucose concentrations were detected by a commercial CGM sensor (Fig. 5A). Across 3 replicates of the same virtual subject and meal scenario, the CGM achieved an average MARD of 3.6% ± 1.0% (*n* = 3) relative to the virtual subject’s glucose reference (Fig. 5A).

**Fig. 5.**
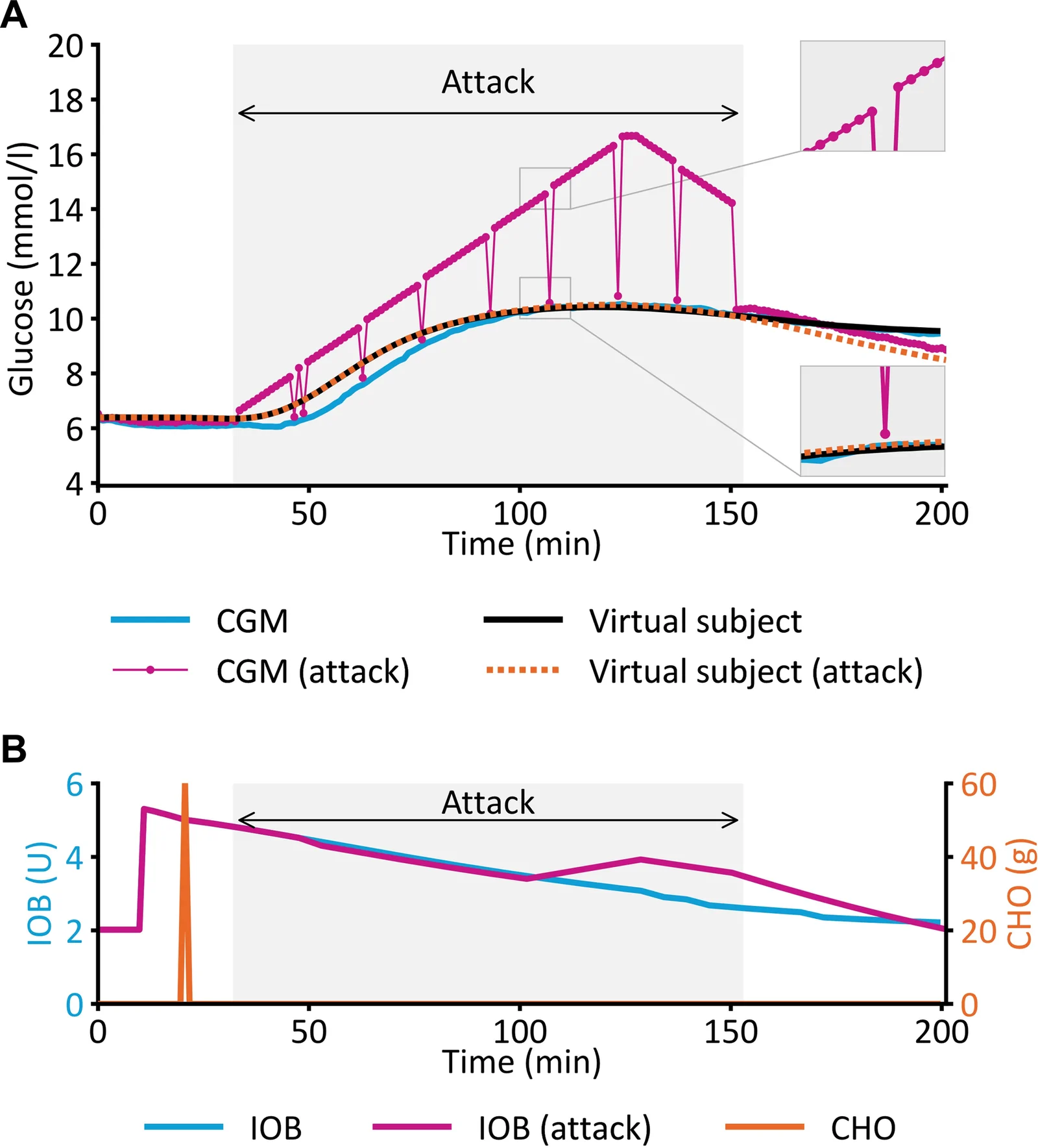
Comparison of baseline and continuous glucose monitor (CGM) attack scenarios. (A) CGM reading and virtual subject glucose level under normal (blue) and attack (magenta) scenarios. (B) Carbohydrate (CHO) intake and insulin on board (IOB) for the virtual subject under normal (blue) and attack (magenta) scenarios. Replicates are included in Figs. S2 and S3.

Prior demonstrations of cyberattacks on CGM include replay attacks [24], bias injection [28], and sensor spoofing [29]. In this study, a hardware-assisted false data injection (FDI) attack scenario was implemented via a smartphone application to tamper with the CGM data. Under normal operation, the sensor detects a glucose reading and stores it locally on the device every minute. During an attack, a custom smartphone application interacts with the CGM to overwrite the true glucose levels with altered data. The specific FDI attack presented here artificially increases the glucose level during a defined attack window. These altered CGM readings are reported to the CGM receiver and further propagated through the AID system, allowing their impact on insulin dosing and subsequent glycemic control to be evaluated (Fig. 5A). Intermittent drops in CGM reading during this scenario reflect transient lapses in the attack, during which real sensor readings were transmitted. Values transmitted during attack lapses correlate with the true glucose concentration in the flow cell, which is set to the virtual subject’s glucose value. The discrepancy between CGM readings and the true virtual participant glucose demonstrate how a cyberattack can impact sensor data integrity.

In response to the manipulated CGM data, the OpenAPS controller’s insulin dosage suggestions increased relative to the baseline scenario, as observed in the spike in insulin around 100 min (Fig. 5B). The CGM MARD during the attack condition increased to 18.8% ± 1.1% (*n* = 3).

## Discussion

This work presents a dynamic HIL test rig for evaluating CGM and AID systems under physiologically realistic and adversarial conditions. The main contribution of this work is a system that preserves real-world dynamics between simulated virtual participants, sensor hardware, and communication pathways between CGM and AID systems. This allows for real-time intervention and interaction, which is an advantage over traditional static penetration testing or predefined scenarios.

### Platform validation and design considerations

A series of validation experiments was conducted to confirm the fidelity of the fluidic system, sensor integration, and real-time physiological coupling prior to closed-loop studies. The objectives were (a) to observe the effects of flow on sensor readings, (b) to evaluate the reproducibility of mixed glucose concentrations, and (c) to determine the system response time.

Flow-dependent effects on glucose sensor readings were characterized using amperometric measurements with premixed glucose solutions. The functionalized SPEs demonstrated glucose responsiveness, although the flow rate affected the magnitude of current readings, necessitating in-system calibration at the operational flow rate. The geometry of commercial CGM sensors—including sensing wire length and hardware attachments—mandates larger millifluidic systems. Physiological interstitial flow rates of 1 to 2 μm/s [30] are impractical for this application, as they would require minutes to hours to refresh the glucose concentration in the flow cell chamber.

While the operational flow rate (47.5 mm^3^/s) is approximately 250 times higher than physiological interstitial flow, they remain within the laminar regime (Re = 3.95 for the aqueous solution at 20 °C flowing through the 5 mm × 19 mm flow cell). Under laminar flow conditions, mixing of the 2 fluid streams from the dual-pump system is minimal. For CGM with small filaments, glucose readings would be highly dependent on the sensor position within the flow cell. A mixing chamber with a magnetic stir bar was designed to address this issue. Although the mixer introduces local fluid velocities higher than the bulk flow, the signal stability observed in both the SPE and commercial CGM filaments confirms that any resulting shear stress remains below the threshold for mechanical or chemical interference damage. While the SPE performed relatively well both with and without the mixer, due to its larger sensing surface area compared to commercial CGM filaments, the signal stability in the presence of the mixer confirms that it enhances the homogeneity and reproducibility of the glucose solution in the flow cell. The mixing chamber may also act as a fluidic capacitor to provide pressure dampening for the pulsatile flow from peristaltic pumps.

A flow rate of 47.5 mm^3^/s was selected based on its demonstrated stability and practical considerations. Faster flow rates enable quicker turnover of solution in the flow cell chamber. However, flow rates beyond 47.5 mm^3^/s would require larger quantities of glucose and buffer solution, posing practical challenges for multihour virtual subject simulations. The system parameters are adjustable, with the flow rate being determined primarily by the constraints of minimum peristaltic pump rates, sensor geometry, and the feasibility of preparing large volumes of solution.

In the presence of the mixing chamber, the system exhibits a minimal time lag of 2.42 ± 0.45 min between when a new glucose concentration is programmed and when the sensor responds to the new concentration (Fig. S1). This meets the design constraint for the test rig is that the time lag should be <5 min. A commercial CGM samples interstitial glucose levels at 1-min intervals and typically performs smoothing functions over intervals of 5 to 15 min. With 3 segments of 50 mm length × 3 mm diameter tubing, a mixing chamber of depth 6 mm and diameter 9 mm, a CGM flow cell with dimensions of 18 × 7 × 5 mm, and an SPE flow cell of dimensions 19 × 19 × 5 mm, the total void volume is approximately 3,900 mm^3^. The void volume alone would account for a 1.5-min lag time at a flow rate of 47.5 mm^3^/s. The remaining 1-min lag time is due to the diffusion and equilibration of the new concentration within the flow cell chamber.

The lag time of the system is within an acceptable range and—as demonstrated by the preprogrammed glucose profiles—does not impede the ability of the test rig to reproduce virtual subject glucose levels. It is important to note that the observed system response time is a function of both the mass transport of new glucose solution from the Y-junction to the sensing region and the in-house sensor response time. Furthermore, delay is inherent in in vivo glucose sensing and closed-loop insulin delivery systems. Thus, the delay associated with glucose transport within the flow cell, sensor response, and communication between the sensor and the virtual participant simulator contributes to the realism of the system and allows the test rig to model how delays may influence responses to cyberattacks.

### HIL integration and significance

The platform addresses a critical gap in medical device development: the absence of standardized methods for evaluating integrated system performance prior to clinical trials. Coupling CGM readings with an adaptation of the UVA/Padova glucose–insulin model and insulin controllers enables the rig to reproduce closed-loop trajectories. The UVA/Padova model includes a virtual population of 300 participants, grouped into children, adolescents, and adults. This enables the test rig to evaluate closed-loop responses across a range of simulated physiological characteristics and participant profiles. The MARD between simulated virtual subject glucose and CGM-measured glucose concentrations on the test rig demonstrates the system’s reliability in HIL operation. This fidelity supports the use of the rig as an intermediate validation step between in silico simulations and human subject testing, potentially shortening the device development cycle and reducing reliance on early-stage clinical trials [31]. As the observed MARD was below 20%, meeting the clinical performance criterion reported in Ref. [32], the results indicate that the flow cell system can provide a sufficiently representative interstitial environment for evaluating CGM performance under physiological glucose conditions. This is consistent with previous flow-based systems that have used electrolyte buffers to interface with commercial CGM [9]. However, one limitation of the present test rig is that it is not designed to assess the physical or mechanical failure modes of CGM, such as compression hypoglycemia, sensor overheating, or sensor detachment from the patient.

### Cybersecurity assessment in AID

Beyond performance validation, the test rig uniquely enables systematic investigation of cybersecurity risks in AID systems. Simulated attacks targeting CGM data integrity resulted in substantially altered insulin-dosing decisions, despite no change in the underlying physiological state, consistent with prior demonstrations of FDI attacks against AID systems [33,34]. The novel cyberattack demonstrated on the CGM was disclosed to the manufacturer.

When the attack lapses, the sensor readings match the true virtual subject glucose, indicating that the sensor is active and continues to accurately read the glucose concentration in the flow cell. This implies that the demonstrated attack overrides the true glucose value without being detected by the sensor firmware or software. Typically, when anomalous or rapidly changing readings are detected, potentially indicating a technical failure, the affected sensors are inactivated. However, this response was not observed in our attack scenario. Instead, the compromised data integrity induces a change in insulin dosing, posing a substantial risk to patient safety. Too much administered insulin could cause hypoglycemia, and suspended insulin could lead to hyperglycemia and diabetic ketoacidosis. Both conditions have life-threatening complications [14]. Although the attack demonstrated in this study artificially increases the glucose concentration relative to the participant’s true physiological state, the same attack method could be adapted to artificially decrease the reported glucose concentration, still potentially leading to inappropriate insulin administration.

These results demonstrate that CGM, often treated as a passive sensing element, constitutes a critical vulnerability within the cyberphysical control loop. The ability to safely simulate such scenarios highlights the importance of integrated resilience testing and motivates the development of anomaly detection and secure communication mechanisms for interoperable medical devices. From a clinical perspective, device interoperability and data aggregation have the potential to improve health and healthcare experiences [35]. However, this must be balanced by standards and regulations to ensure that interoperable and interconnected systems are robust and resilient [36].

Existing standards for CGM and AID systems include interoperability standards between CGM and personal mobile devices (IEEE P11073-10425) [37] and interoperable standards and designations by the Food and Drug Administration (FDA). Within the AID system, the FDA has regulations for integrated continuous glucose monitors (21 CFR 862.1355), interoperable automated glycemic controllers (21 CFR 862.1356), and interoperable alternate controller-enabled infusion pumps (21 CFR 880.5730). Despite the existence of these regulations, our results demonstrate that vulnerabilities still exist in CGM and AID. This underscores the importance of having a standardized testing platform, such as this, to systematically validate device performance against published regulations.

### Platform utility and future applications

This work introduces a dynamic glucose sensor test rig capable of reproducing biorealistic interstitial glucose fluctuations in real time. By integrating a fluidic system, commercial CGM, a real-time glucose–insulin response simulator, and an insulin controller, the system establishes an HIL environment for evaluating AID systems under both physiological and adversarial conditions. The test rig provides a modular, scalable, and reproducible platform for testing CGM, AID controllers, and cybersecurity countermeasures. The findings have implications for both clinical safety and medical device cybersecurity, as the demonstrated cyberattack can alter insulin dosing, highlighting how cybersecurity vulnerabilities in closed-loop therapeutics can translate to risks to patient safety. These findings emphasize the need for cybersecurity considerations to be integrated into the development, validation, and clinical evaluation of AID systems.

While the test rig does not replace human trials, it provides an intermediate validation step that can shorten development cycles, reduce the risk of early design failures, and systematically evaluate protective measures in adversarial scenarios. Future work will investigate other modes of attack in AID, incorporate active anomaly detection to reduce adversaries’ success rate, and extend the approach to other classes of wearable biosensors.

This test rig therefore addresses a risk that extends beyond conventional algorithmic output risk, where automated software decisions could cause harm through misrepresentation, overreliance, or misuse of results. AID systems are specifically designed to minimize patient interaction, thereby reducing the potential for patient error in interpreting glucose readings or calculating insulin doses. In the case of a CGM cyberattack, the insulin control algorithm continues to operate as designed but makes decisions based on compromised data inputs. Furthermore, AID systems undergo rigorous safety testing and validation. Therefore, patients are not blindly trusting the devices—they are rightly relying on prescribed and validated medical technologies. While safety has always been the cornerstone of regulatory approval processes, the role that security plays in safety has only recently been explored. A deliberate, malicious cyberattack that compromises otherwise correctly functioning automated systems presents a distinct risk from errors in automated software outputs and requires complementary mitigation strategies, including anomaly detection and hardware-based security mechanisms.

Finally, while we use CGM and AID systems as an exemplar application, the concept can generalize to other wearable and point-of-care sensors where the pharmacokinetics or physiological dynamics of the target analyte are sufficiently understood (for example, lactate, ethanol in sweat, or transdermal drug concentrations). A standardized, automated, and reproducible HIL platform can therefore function as an extensible test bed for the next generation of secure, connected biosensors, ultimately accelerating development cycles, improving preclinical evaluation, and strengthening the evidence base required by regulators and clinicians.

A dynamic HIL test rig is the definitive cornerstone for assessing the security of CGM and AID systems because it bridges the gap between digital exploitation and life-critical physiological impact. By integrating actual medical hardware with real-time physiological simulators, researchers can move beyond theoretical vulnerabilities to quantify the exact medical consequences of an attack—such as how manipulated CGM data translate into changes in insulin dosage. This environment is essential for testing the control algorithm’s resilience against adversarial attacks, such as signal injections designed to bypass safety alarms. Future work could integrate insulin pump HIL to provide more comprehensive coverage of the AID system’s cybersecurity attack surface. Ultimately, an HIL rig provides the high-fidelity evidence required to validate that a device will be fail-safe under cyber-adversity.

## Materials and Methods

### Materials

*m*-Phenylenediamine (mPD), glucose oxidase (from *Aspergillus niger*), poly(ethylene glycol) diglycidyl ether (average *M*_N_ 500, PEG-DE), bovine serum albumin, and glycerol were purchased from Sigma-Aldrich. Platinum (DRP-550BT) SPEs were purchased from Metrohm DropSens. Electrochemistry experiments were performed using a PalmSens potentiostat (PalmSens4, PalmSens BV).

### Glucose–insulin response simulator

A real-time implementation of the UVA/Padova model was developed to make it responsive to real-time CGM data for the HIL system. The accuracy of the real-time stepwise simulator was validated by comparing to outputs from the UVA/Padova model. Simulations were run across 30 virtual subjects (including children, adolescents, and adults) under 3 meal scenarios (90 total cases). To ensure consistency, initial conditions were identical between the real-time stepwise and UVA/Padova implementation, and meals of the same quantity were delivered at the same simulation time steps. The RMSE of the glucose level was computed for each simulation pair.

$$\text{RMSE}=\sqrt{\frac{\sum_{j=1}^{J}{\left(y_{j}-{\hat{y}}_{j}\right)}^{2}}{J}}$$ (1)

where $y_{j}$ is the glucose value at step j for the UVA/Padova simulator and ${\hat{y}}_{j}$ is the glucose value at step j for the real-time simulator across J time steps.

The MARD was also computed for simulation pairs [25], using the UVA/Padova value as reference and replacing CGM with real-time simulator glucose data:

$$\text{MARD}=\frac{1}{J}\sum_{j=1}^{J}\left|\frac{{\text{CGM}}_{j}-{\text{Reference}}_{j}}{{\text{Reference}}_{j}}\right|$$ (2)

where ${\text{CGM}}_{j}$ is the glucose value at step j for the real-time simulator and ${\text{Reference}}_{j}$ is the glucose value at step j for the UVA/Padova simulator across J time steps.

### Implementation of the insulin controller

The insulin control algorithm selected for use in this proof-of-concept system was an open-source artificial pancreas system (OpenAPS). The basal and bolus amounts are determined by predefined equations in OpenAPS. The inputs to the equations are real or simulated CGM values, subject data, meal schedule, and previously administered acting insulin. Control parameters for OpenAPS were optimized for individual virtual subjects, with a target of achieving 76.1% ± 12.1% TIR (3.9 to 10.0 mmol/l), 23.8% ± 9.7% TAR (>10.0 mmol/l), and near 0% TBR (<3.9 mmol/l) for adults and 69.6% ± 11.7% TIR, 30.4% ± 9.1% TAR, and near 0% TBR for children, to mimic the performance of OpenAPS in real patients [38]. Referring to Burnside et al. [38], the extremely high hyperglycemia (13.9 mmol/l) TIR is merged in the high hyperglycemia (10.0 to 13.9 mmol/l). The performance of the OpenAPS implementation was validated by comparing the TIR, TAR, and TBR to the results from the UVA/Padova model with in-built basal–bolus insulin controller.

### Glucose solution and interstitial fluid matrix

Artificial interstitial fluid buffer was prepared to mimic the environment in which CGM sensors operate. There are differences in the electrolyte concentrations of intracellular fluid, interstitial fluid (part of the extracellular fluid), and blood. Interstitial fluid has approximately 140 mM Na^+^, 115 mM Cl^−^, 30 mM HCO_3_^−^, 3.8 mM K^+^, 1 mM HPO_4_^2−^, and 0.8 mM Mg^2+^ [39]. Thus, an artificial matrix buffer containing 115 mM NaCl, 30 mM NaHCO_3_, 1 mM K_2_HPO_4_, and 0.8 mM MgCl_2_ was prepared and titrated to pH 7.4 to 7.5 with HCl. Glucose solution was prepared as a 1 M or 50 mM stock solution in interstitial fluid buffer.

### Preparation of enzymatic glucose electrodes

SPEs were modified to be glucose sensitive using a method adapted from Vasylieva et al. [40,41]. Briefly, the SPE on-chip Ag reference was chlorinated by applying a +0.24-V potential for 15 min against a commercial Ag|AgCl–1 M KCl reference electrode and a platinum coil auxiliary electrode. Next, mPD was electropolymerized on the SPE platinum working electrode by applying a +0.7-V potential for 20 min against on-chip reference and counter electrodes. Successful deposition was validated by comparison of the cyclic voltammograms of the [Fe(CN)_6_]^3+^/[Fe(CN)_6_]^4+^ redox probe before and after electropolymerization. A glucose oxidase hydrogel was prepared, consisting of 60 mg/ml glucose oxidase, 30 mg/ml bovine serum albumin, 1% v/v glycerol, and 14.82 mg/ml PEG-DE in 1× phosphate-buffered saline (100 μl); 10 μl was drop-casted onto the mPD-coated SPEs and oven-dried at 55 °C for 2 h. The prepared glucose electrodes were stored in the freezer and hydrated in buffer for 3 h prior to use.

### Electrochemical measurements

Amperometry measurement with the glucose oxidase-functionalized electrodes was performed at room temperature using a 3-electrode setup with the on-chip reference electrode and counter electrodes. The working electrode was held at +0.7 V relative to the reference. The sensitivity and dynamic range of the electrode were evaluated by measuring the steady-state faradaic current in varied glucose concentrations to generate multipoint calibration plots.

### Flow cells

Flow cells consist of a central chamber with inlet and outlet channels (Fig. 6). Chamber depth was optimized to ensure full immersion of the sensing element while minimizing void volume (for faster turnover of solution in the chamber). All flow cells were fabricated via 3D printing (SLA transparent resin). As the sensing element is at the top surface, the chamber was printed open and sealed with a thin-film well plate seal. Punctures were made in the thin films to insert sensors, which were further sealed with thermoplastic glue. Flow performance was evaluated under variable pump rates to observe the effect on sensor readings and equilibration time.

**Fig. 6.**
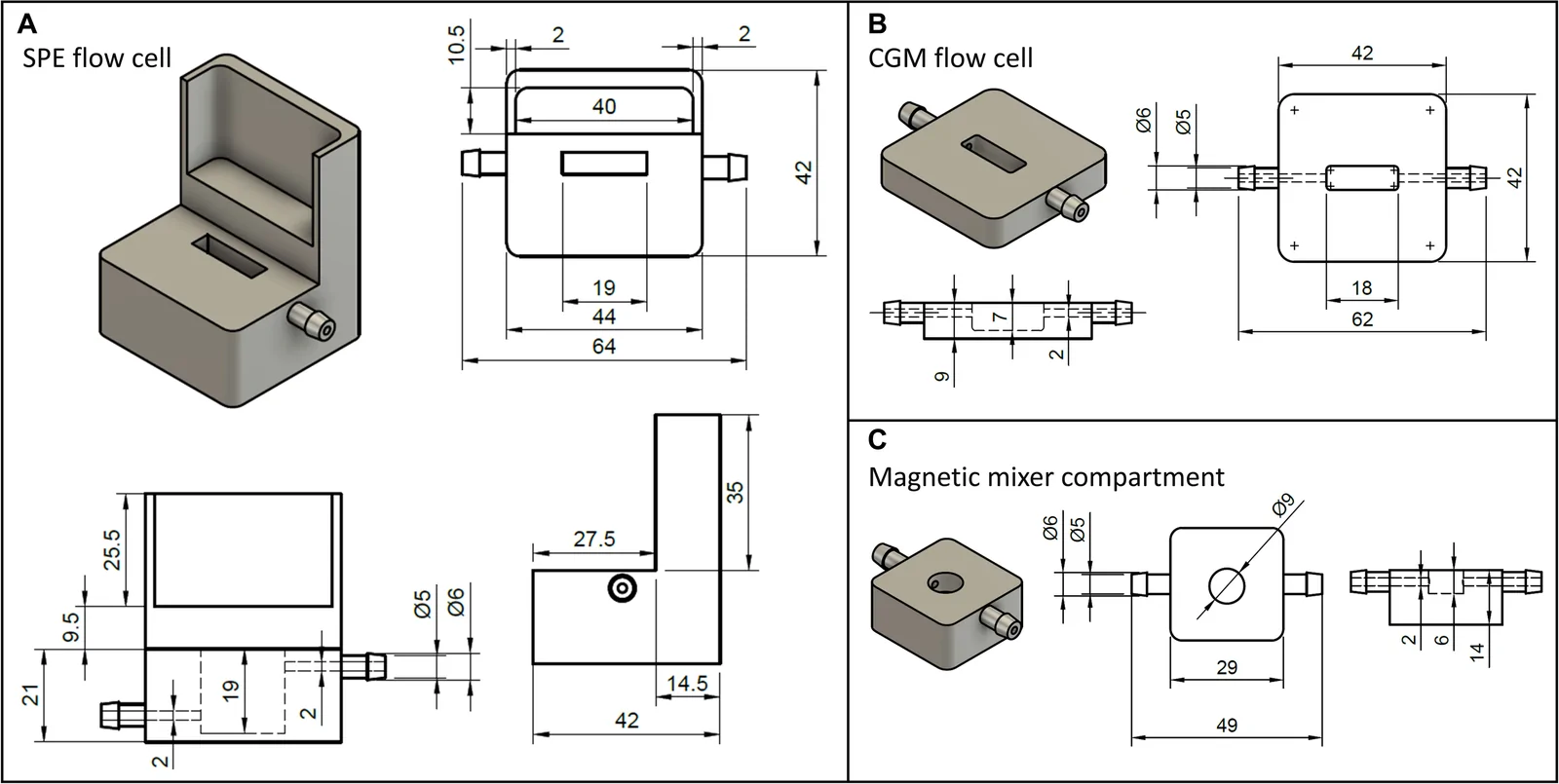
Modular flow cell designs for (A) screen-printed electrode (SPE) glucose sensors, (B) a commercial continuous glucose monitor (CGM), and (C) a magnetic stir bar mixing compartment. Dimensions are in millimeters (mm). Channel openings were sealed with thin-film adhesives. Punctures were made in the thin films to insert sensors, which were further sealed with thermoplastic glue. The SPE sensor was positioned vertically and attached to a Metrohm SPE DropSens box connector. The 40 mm × 9.5 mm ledge in flow cell A is specifically designed to support the Metrohm SPE connector and position the SPE centrally within the flow chamber.

### Flow system design

Figure 1 illustrates the architecture of the dynamic glucose sensor test rig. Two computer-controlled peristaltic pumps draw liquid from either a glucose stock solution reservoir or a buffer solution reservoir. By adjusting the ratio of the 2 flow rates while maintaining a constant total flow rate, the system generates user-defined glucose concentrations within a custom-3D-printed flow cell (Fig. 6). An in-house glucose-sensitive SPE or a commercial CGM was mounted directly onto a flow cell chamber, ensuring continuous exposure of the sensing element to dynamically varying glucose concentrations. Where indicated, a magnetic stirrer mixing chamber was placed upstream of the glucose sensors. The SPE readings were collected by amperometry as described above, and glucose concentration was determined from the calibration plot. The CGM readings were transmitted via a smartphone application.

### Validation

#### Flow-rate effects on sensors

A premixed glucose solution of 6 mmol/l was prepared. An in-house SPE glucose sensor was attached into an SPE flow cell. The SPE flow cell was connected to a single peristaltic pump, and the constant concentration glucose solution was pumped into the flow cell at varying linear flow rates of 0.025, 0.05, 0.1, 0.25, and 0.5 mm/s. The pumps were turned on for 30 min at a given flow rate and then turned off for 30 min. Amperometry data were collected.

#### System calibration

To calibrate the amperometry readings, premixed glucose solutions (0 to 12 mmol/l) were prepared. An operating flow rate of *Q* = 47.5 mm^3^/s was selected for the system. An SPE was fixed into a flow cell and connected to a single peristaltic pump. A single premixed glucose solution was pumped through the flow cell for 20 min, and the process was repeated for different glucose solutions (0 to 12 mmol/l) to create a calibration curve relating current and glucose.

To verify the ability to achieve a set glucose value from mixing the output of the 2 peristaltic pumps, the SPE flow cell was then attached to both peristaltic pumps via a Y-connector. Using the pump control script, a glucose concentration was set and run for 20 min before changing stepwise to the next concentration. To obtain a set glucose concentration, the pump control program varies the ratio of glucose pump and buffer pump flow rates while maintaining total constant flow rate, in this case 47.5 mm^3^/s. Subsequently, the program sends a signal to the pumps via USB, setting the revolutions per minute (RPM) corresponding to the flow rate. This experiment was repeated with and without the presence of a magnetic mixing chamber preceding the sensor flow cell. Commercial CGM sensors do not perform well in stepwise calibration due to a max rate of change restriction and thus were not deployed in these validation experiments.

#### Dynamic glucose trajectory

A sample glucose trajectory dataset was generated from the real-time glucose–insulin simulator and preprogrammed into the pump controller. This was then used to demonstrate the flow system’s ability to generate a realistic glucose profile, as measured by the glucose sensors. A commercial CGM flow cell and SPE flow cell were connected in series, downstream of the magnetic mixing chamber. The blood glucose values from the pregenerated profile were passed to the pump flow system once per minute, dynamically changing the concentration in the flow cells. The MARD was calculated using the programmed glucose as reference compared to either the SPE or CGM sensor readings.

### Design of the simulated hybrid closed-loop system

The real-time glucose–insulin response simulator with an HIL CGM fluidic system was integrated with an insulin controller to create an AID loop. Once per minute, the backend platform, which controls the operation of the cyberphysiological test rig system, triggers an action in the virtual subject and OpenAPS. The backend transmits CGM data to the virtual subject, which are further passed to OpenAPS. OpenAPS then determines the appropriate insulin dose based on the CGM value, meal scenarios, and predefined state variables. This dose is returned into the glucose–insulin response simulator, which predicts the glucose concentration at the next time step based on the insulin dose and the true blood glucose (Fig. S4). It is important to note that the internally stored true blood glucose is distinct from CGM readings. This is representative of the real-world system, in which CGM sensors are reporting the user’s glucose level.

The updated glucose value for the next time step is then sent to the pump-rate calculator, which converts this target concentration into a ratio of glucose-to-buffer flow. Flow rates are converted to pump RPM using a predetermined calibration curve, and serial commands are sent to the pumps via USB. The hybrid closed-loop model updates the glucose value once per minute, reflective of the time resolution of typical CGM (Fig. 1).

### Front end

A graphical front end enables users to configure virtual subject parameters (insulin sensitivity, carbohydrate ratios, and meals) and physical test rig settings (flow cell geometry, reservoir concentrations, and pump calibration variables). During operation, the interface displays real-time CGM measurements, true virtual subject glucose levels, insulin dosages, and pump commands. The CGM readings are retrieved via a smartphone application, and all data from the simulations are logged in a .csv file.

### Running virtual scenarios

To validate the performance of the hybrid closed-loop test rig, a commercial CGM was attached to the flow cell, and the simulation was initialized. A single meal scenario (60 g) for adult subject #007 was repeated on the test rig 3 times. The MARD was calculated for all simulations using the CGM data with virtual subject simulator glucose data as reference. To evaluate cybersecurity vulnerabilities, CGM sensors were subjected to synthetic attack scenarios. The impact on glycemic control was assessed by monitoring simulated insulin-dosing decisions and virtual subject glucose trajectories.

## Data Availability

All raw data files are available on the Imperial College London institutional repository. The source code is available at https://github.com/ImperialGlobalSingapore/simglucose.
