# Supplementary material for: Toward Trustworthy Diabetes Technologies: A Dynamic Platform for Safety and Security Evaluation
Source: BME Front. 2026 Sep 30;7:0317. doi: 10.34133/bmef.0317 (PMC13624366; doi:10.34133/bmef.0317)
Supplement: Supplementary file 1 [file bmef.0317.f1.pdf]

# Supplementary Materials: Toward trustworthy diabetes technologies: a dynamic platform for safety and security evaluation

**Anna R. Bird[1*+], Vivek Mohan[1,2*+], Kexin Wei[1], Zong Cao[1,3], Shi Hoe Ng[1], Manasvi M. Mulay[1,4], Viktor Schlegel[1,5,6], Miro G. Moffett[1], James E. Moore Jr[1,5], Chip-Hong Chang[2], Nick Oliver[1,7], and Anil A. Bharath[1,5]**

[1]Imperial Global Singapore, Imperial College London, Singapore, Singapore
[2]School of Electrical and Electronic Engineering, Nanyang Technological University, Singapore, Singapore
[3]College of Computing and Data Science, Nanyang Technological University, Singapore, Singapore
[4]School of Chemistry, Chemical Engineering, and Biotechnology, Nanyang Technological University, Singapore, Singapore
[5]Department of Bioengineering, Imperial College London, London, United Kingdom
[6]The University of Manchester, Manchester, United Kingdom
[7]Department of Metabolism, Digestion and Reproduction, Imperial College London, London, United Kingdom
[*]a.bird@imperial.ac.uk, v.mohan@imperial.ac.uk
[+]these authors contributed equally to this work

## ABSTRACT

Automated insulin delivery (AID) systems depend on continuous glucose monitoring (CGM) as their primary sensing input, making evaluation of the accuracy, robustness, and security of these sensors essential for safe diabetes management. However, there are limited assessment tools which preserve the communication and timing paths of CGM sensors within an AID system. We present a modular hardware-in-the-loop (HIL) platform that reproduces physiological glucose dynamics, bridging the gap between simulation and clinical validation. The system integrates programmable peristaltic pumps, 3D-printed flow cells, glucose sensors, and a real-time virtual subject simulator. The platform generates reproducible glucose trajectories and supports closed-loop operation through integration with an open-source insulin controller. To demonstrate the significance of this platform, we use it to simulate CGM cyberattacks that produce inappropriate insulin dosing in the virtual subject. This HIL platform provides a reproducible, physiologically grounded test environment for AID safety and security assessment.

**Acknowledgement:** This work was supported by the National Research Foundation, Prime Minister's Office, Singapore under its Campus for Research Excellence and Technological Enterprise (CREATE) - IN-CYPHER programme.

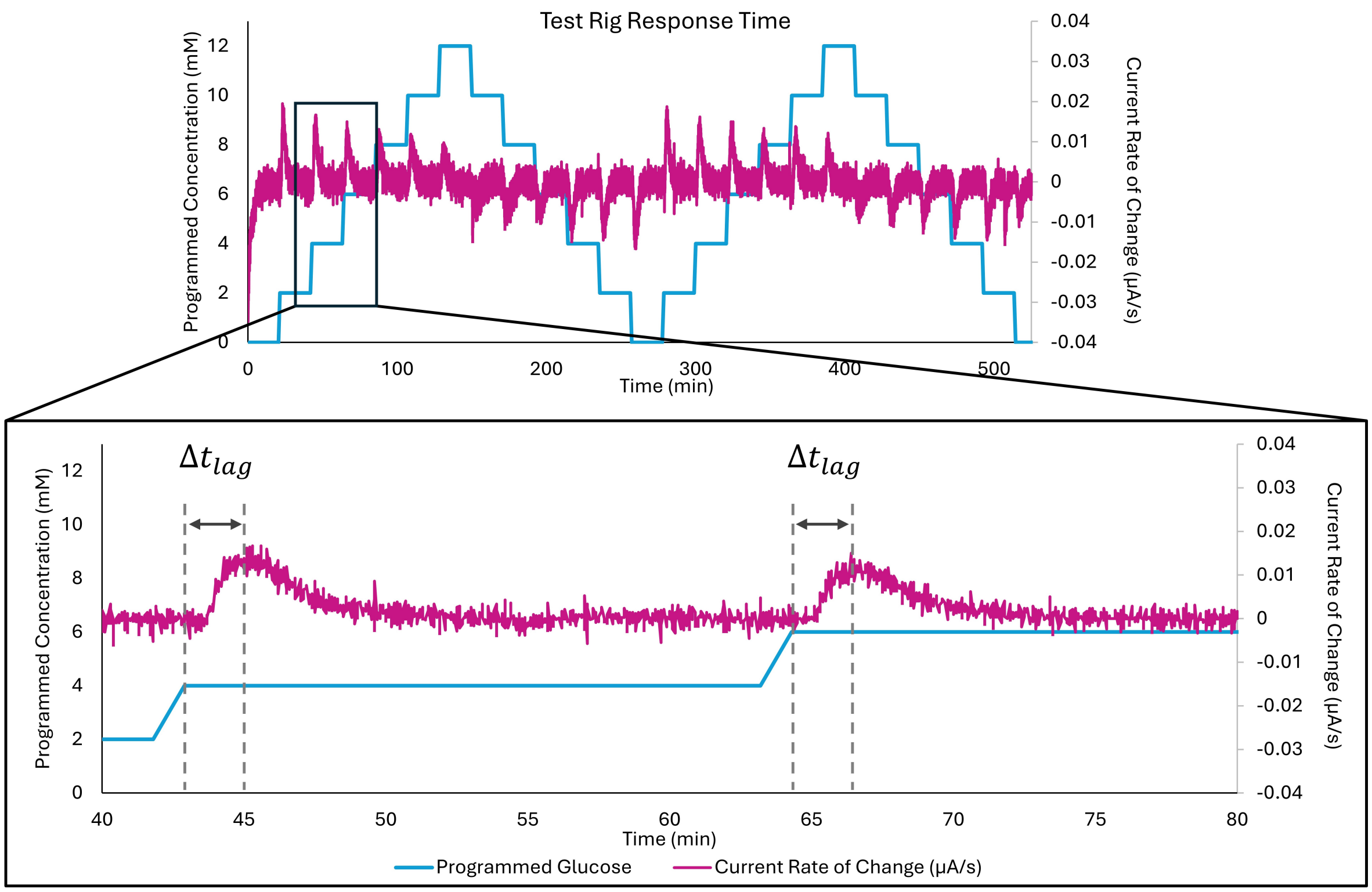


**Figure S1.** The response time of the sensor in the fluidic test rig, as determined by the derivative of current signal with respect to time (magenta) and the glucose concentration programmed by the pump controller (blue). The time lag was calculated from the difference between when the programmed glucose concentration was changed, and when the derivative of current with respect to time reached a local maximum (increasing concentration) or local minimum (decreasing concentration).

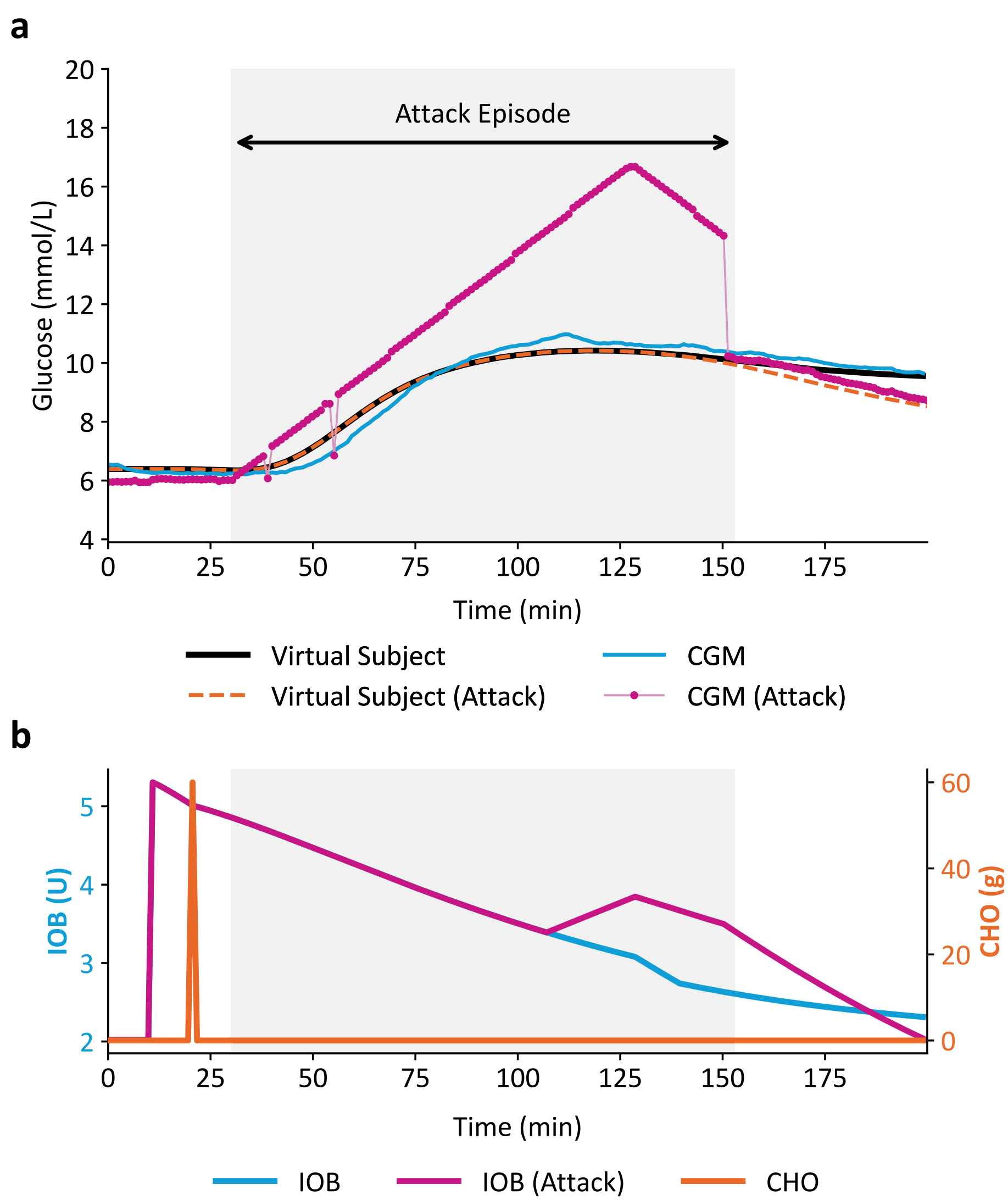


**Figure S2.** Second replicate of closed-loop simulations with and without attack. **a** CGM response and virtual participant response under normal and attack scenarios. **b** Carbohydrate (CHO) intake and insulin doses delivered to virtual participant, under normal and attack scenarios.

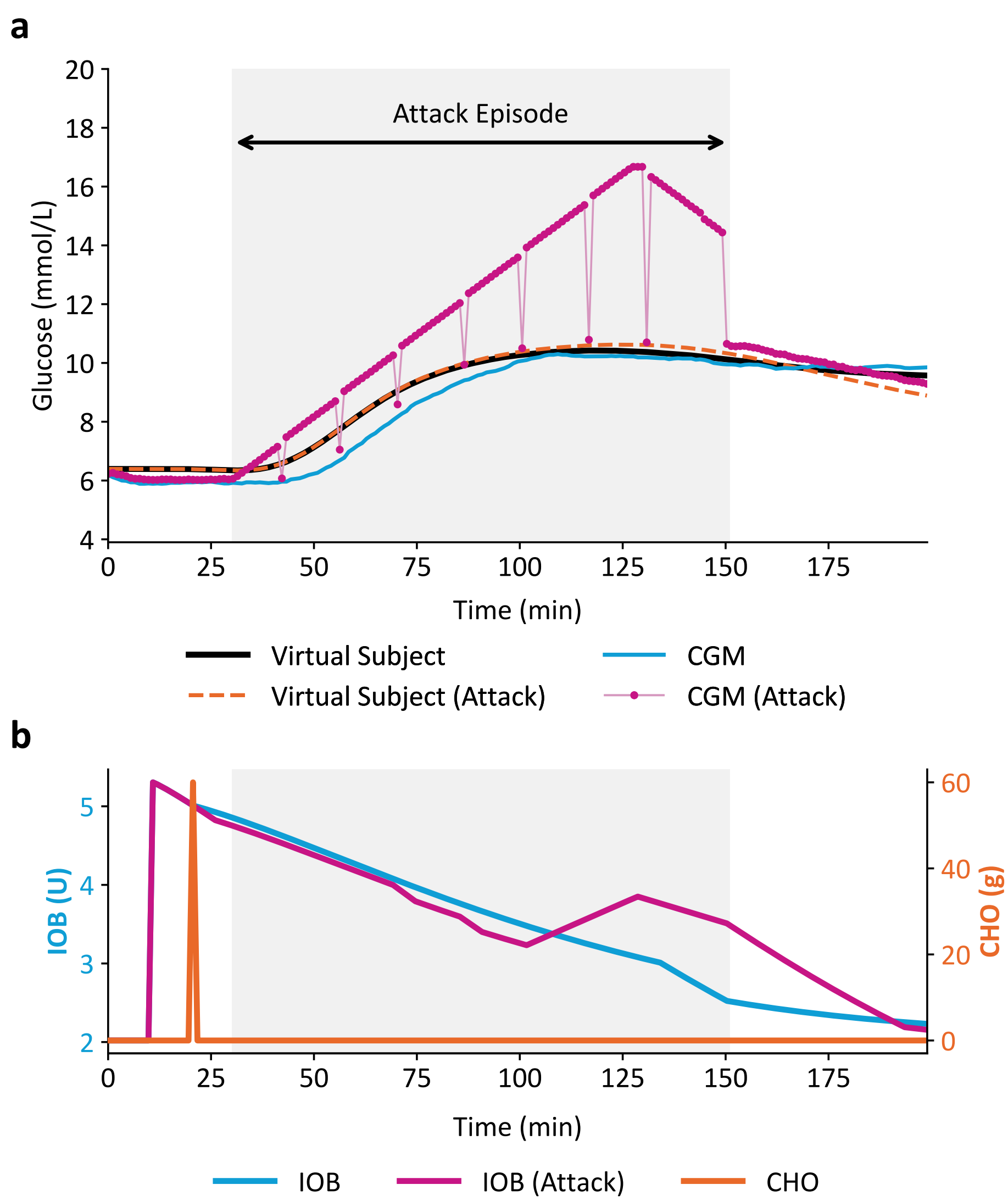


**Figure S3.** Third replicate of closed-loop simulations with and without attack. **a** CGM response and virtual participant response under normal and attack scenarios. **b** Carbohydrate (CHO) intake and insulin doses delivered to virtual participant, under normal and attack scenarios.

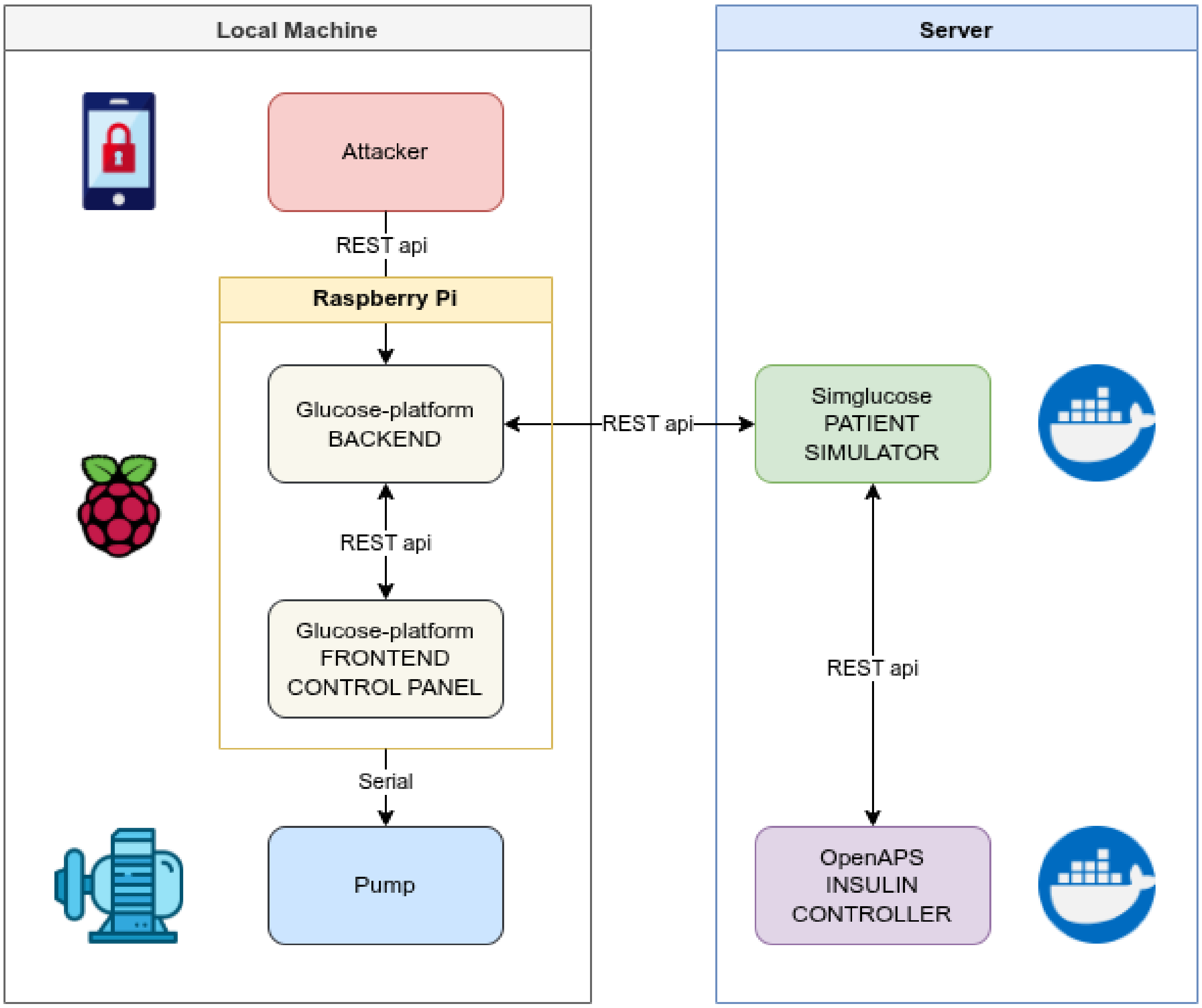


**Figure S4.** Data communication pathways between frontend, backend, and servers
